# Sulforaphane Modulates Joint Inflammation in a Murine Model of Complete Freund’s Adjuvant-Induced Mono-Arthritis

**DOI:** 10.3390/molecules23050988

**Published:** 2018-04-24

**Authors:** João Francisco Silva Rodrigues, Cristiane Santos Silva e Silva Figueiredo, Thayanne França Muniz, Alana Fernanda Silva de Aquino, Larissa Neuza da Silva Nina, Nagila Caroline Fialho Sousa, Luis Claudio Nascimento da Silva, Breno Glaessner Gomes Fernandes de Souza, Tatiane Aranha da Penha-Silva, Ana Lúcia Abreu-Silva, Joicy Cortez de Sá, Elizabeth Soares Fernandes, Marcos Augusto Grigolin Grisotto

**Affiliations:** 1 Post-Graduation Program, Uniceuma University, são Luis 65075, MA, Brazil; joaofranciscosr@hotmail.com (J.F.S.R.); cristianeloud@gmail.com (C.S.S.e.S.F.); thayanne_muniz@hotmail.com (T.F.M.); fernanda.aquino2@hotmail.com (A.F.S.d.A.); l.snina.lnina@gmail.com (L.N.d.S.N.); nagila-caroline2011@live.com (N.C.F.S.); luisclaudionsilva@yahoo.com.br (L.C.N.d.S.); joicyvet@hotmail.com (J.C.d.S.); lizbeth_fernandes@yahoo.co.uk (E.S.F.); 2 Florence Institute, Imunology Departament São Luis 65075, Brazil; 3 Pathology Departament, State University of Maranhao, Sao Luis 65075, MA, Brazil; brenoggfs@hotmail.com (B.G.G.F.d.S.); tatianearanha@hotmail.com (T.A.d.P.-S.); abreusilva.ana@gmail.com (A.L.A.-S.)

**Keywords:** rheumatoid arthritis, sulforaphane, oedema, IL-6, thioredoxin reductase

## Abstract

Rheumatoid arthritis (RA) is characterized by inflammation of one or more joints, and affects ~1% of the adult population worldwide. Sulforaphane (SFN) is a natural compound that has been suggested as an antioxidant. Here, SFN’s effects were evaluated in a murine mono-arthritis model. Mono-arthritis was induced in mice by a single intra-articular injection of Complete Freund’s Adjuvant (CFA-10 µg/joint, in 10 µL) into the ipsilateral joint. The contralateral joint received an equal volume of PBS. On the 4th day post-joint inflammation induction, animals received either SFN (10 mg/kg) or vehicle (3% DMSO in saline), intraperitoneally (i.p.), twice a day for 3 days. Joint swelling and secondary mechanical allodynia and hyperalgesia were evaluated over 7 days post-CFA. After this period, animals were culled and their blood and synovial fluid samples were collected for analysis of cell populations, cytokine release and thioredoxin reductase (TrxR) activity. Knee joint samples were also collected for histology. SFN reduced joint swelling and damage whilst increasing the recruitment of Ly6C^+^ and Ly6G^+^ cells to CFA-injected joints. SFN-treated animals presented down-regulation of CD11b and CD62L on synovial fluid Ly6G^+^ cells. Synovial fluid samples obtained from CFA-injected joints and plasma samples of SFN-treated mice presented higher levels of IL-6 and increased activity of TrxR, in comparison with controls. These results indicate that SFN reduces knee joint damage by modulating cell activation/migration to the joints, cytokine production and increasing the activity of TrxR, and therefore, may represent an alternative treatment to joint inflammation.

## 1. Introduction

Rheumatoid arthritis (RA) affects ~1% of the adult population, with a female to male ratio of 3:1. It is defined as the inflammation of one or more joints, with the presence of pain, swelling, erythema, an increase in the temperature of the affected areas [1]. Importantly, RA can cause loss of function or stiffness of the affected joints diminishing life quality. The inflammatory process in RA includes the presence of autoantibodies [2], synovial hyperplasia, angiogenesis and bone loss [3]. Although these inflammatory changes have been extensively investigated, their trigger mechanisms are not fully understood [4]. During RA, there is an ongoing infiltration in to the synovial space [5,6], and a timely and intense release of pro-inflammatory cytokines such as IL-1, IL-6, TNF-α and chemokines [7]. Increased oxidative stress also occurs, culminating with the generation of reactive oxygen (ROS) and nitrogen (RNS) species [8]. All these events contribute to cartilage damage [9] and intensify the joint inflammation and sensitization of neuronal afferents leading to a chronic state of inflammatory pain [10]. Although current therapies have substantially improved patient’s life quality by attenuating pain and inflammation [11,12,13], they do not recover the joints from previous damage made and are note always effective in stopping disease progress [14,15,16].

Sulforaphane (SFN) is a natural isothiocyanate found in plant species belonging to the Brassicaceae family such as broccoli, cabbage and brussel sprouts [17]. SFN’s protective therapeutic actions were previously investigated in renal fibrosis [18], cancer [19,20], renal and intestinal damage caused by ischemia-reperfusion [21,22] and angiogenesis [23]. These effects have been associated with SFN ability to activate antioxidant pathways including the thioredoxin reductase (TrxR) system and to induce the production of cytoprotective proteins [24,25,26]. Indeed, SFN is a potent activator of Nrf2 [27], a transcription factor involved in the expression of a range of genes associated with the cellular antioxidant and anti-inflammatory defence [28]. Also, a role for SFN as a scavenger of superoxide was recently described; however, this effect is not yet completely understood [29]. SFN was previously shown to inhibit synovial hyperplasia and T cell proliferation, and to decrease IL-17 and TNFα production by T cells [30]. The same study also demonstrated that SFN attenuates the severity of experimental RA and the production of auto-antibodies. However, little is known of SFN ability to reduce pain and neither of its ability to regulate cytokine and oxidative stress generation in joint inflammation. Here, we investigated the effects of SFN in a mouse model of Complete Freund’s Adjuvant (CFA)-induced mono-arthritis. We found that SFN attenuates joint damage by modulating antioxidant pathways, cytokine production and cell activation/migration to the joints.

## 2. Results

### 2.1. SFN Reduces Knee Joint Swelling but Not Nociception in Animals with CFA-Induced Joint Inflammation

**Figure 1:**
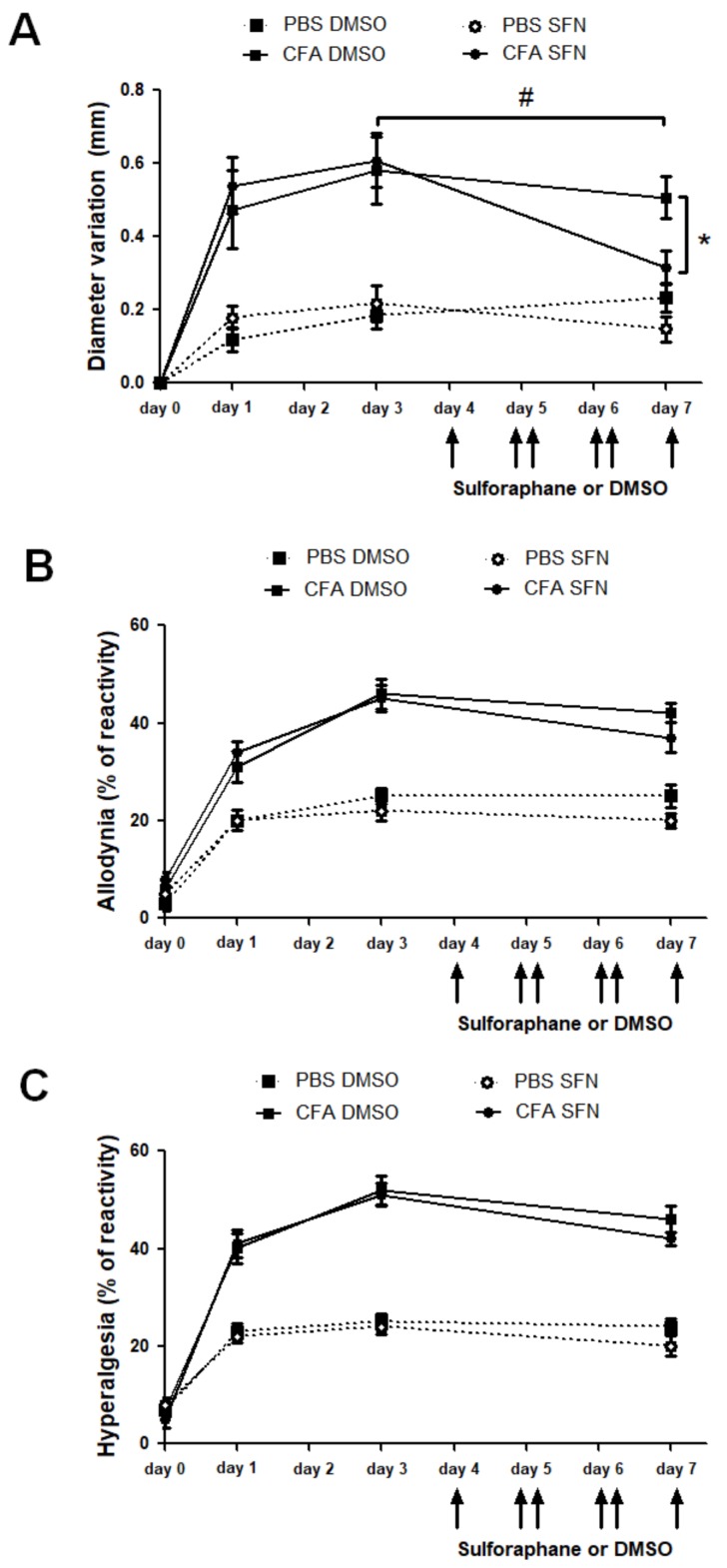
Effects of sulforaphane in the mechanical nociception and knee joint thickness of mice with mono-arthritis. Injection of CFA (10 μL ipsilateral knee) and PBS (10 μL in the contralateral knee) was performed at day 0. (**A**) Variation (mm) of knee diameter (joint oedema) was measured at days 0 (prior to injection), 1, 3 and 7. Secondary allodynia (**B**) and mechanical hyperalgesia (**C**) were measured by percentage of paw withdrawal reflex relative to stimulation with Von Frey filaments 0.4 and 0.6 g, respectively. Six consecutive doses of SFN or DMSO were injected i.p. every 12 h starting at 7:00 a.m. on 4th day. Data are expressed as mean ± SEM. Experiments were performed in four different occasions (*n* = 14 animals per group). * # *p* < 0.05.

Joint inflammation is characterized by increased diameter (indicative of joint oedema) and nociception. Here, CFA was used to trigger joint inflammation in the ipsilateral joint whilst the contralateral side was used as a control within the same animal. As expected, CFA injection caused a marked oedema in the ipsilateral joint which remained present over 3 days. Following 4 days of SFN treatment, mice exhibited reduction in knee swelling in their CFA-injected joints in comparison with the measurements taken on day 3 and also with the knee diameter of vehicle-treated animals (Figure 1A). The swelling in PBS (contralateral)-injected joints was similar in vehicle- and SFN-treated mice, ranging from 0.1 to 0.2 mm over the observation period (Figure 1A).

CFA induced bilateral secondary hyperalgesia and allodynia as mice presented increased reactivity in both ipsilateral and the contralateral paws. Both nociceptive responses remained elevated through the 7-day time course (Figure 1B,C). Treatment with SFN did not alter this response as reactivity in this group was found to be similar to that observed in vehicle-treated mice (Figure 1B,C).

### 2.2. SFN Attenuates the Joint Inflammation/Damage Caused by Intra Articular CFA-Injection

**Figure 2:**
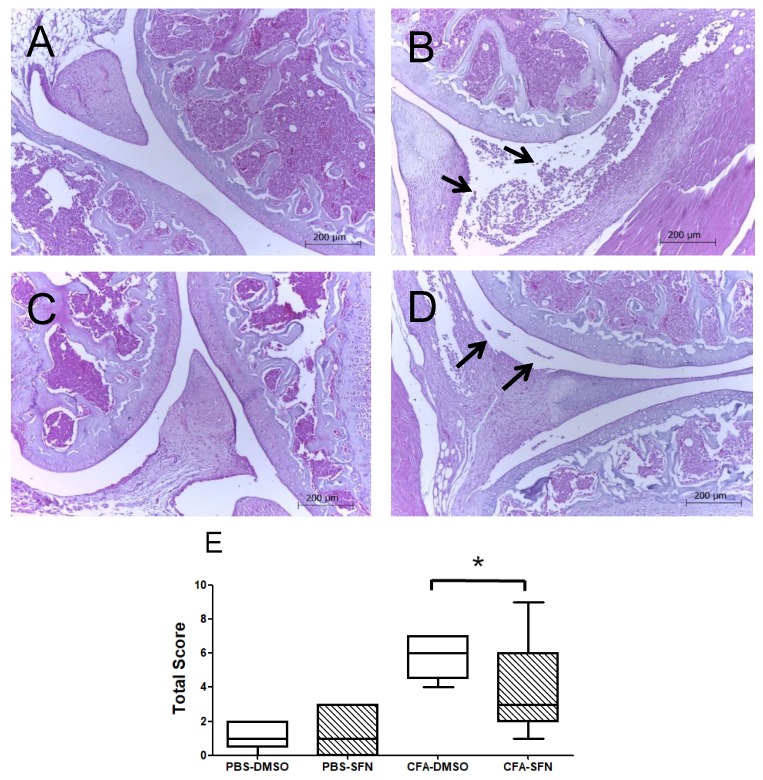
Effects of sulforaphane in attenuation of joint inflammation/damage caused by intra articular CFA-injection. Representative histologic sections of knee joints collected from animals with CFA-induced mono-arthritis (*n* = 5/group). Panels: (**A**) morphological aspect of saline-injected joints treated with vehicle (DMSO), (**B**) morphological aspect of CFA-injected joints treated with vehicle (DMSO), (**C**) morphological aspect of saline-injected joints treated with SFN and (**D**) morphological aspect of CFA-injected joints treated with SFN. Arrows indicate the presence of cellular infiltrates in the synovial space of CFA joints. Sections were stained with hematoxylin and eosin. (**E**) Represents the sum of a 0–3-point score of knee joint inflammation scale used for each parameter: Synovial hypertrophy, cellular infiltration, cartilage destruction, bone erosion (0-normal, 1-mild, 2-moderate, 3-severe). The score of joint inflammation was denoted by the median (minimum-maximum) values. Experiments were performed with 5 animals per group. * *p* < 0.05.

Figure 2 depicts the results obtained in the histologic analysis of knee joints collected from vehicle- and SFN-treated animals (*n* = 5/group). Panels A–D indicate the morphological changes observed for saline and CFA-injected joints of both groups. 

The arrows indicate the presence of cellular infiltrates in the synovial space of CFA joints, which was more intense in those obtained from animals administered with vehicle in comparison with the SFN group (Panels B and D, respectively). Similarly, the CFA joints of animals treated with SFN exhibited less synovial hypertrophy, absence or lower intensity of cartilage damage and bone erosion in their tibiofemoral joints in comparison with the vehicle group. Overall, the CFA-injected joints of SFN-treated mice presented with a smaller score of joint inflammation (Figure 2E) as denoted by the median (minimum-maximum) values, than those of vehicle-treated animals: 6.0 (4.0–7.0) for vehicle CFA and 3.0 (1.0–9.0) for sulforaphane CFA. No differences were observed between saline-treated joints of vehicle- and SFN-administered mice. Registered values for vehicle saline and SFN saline joints were 1.0 (0.0.–2.0) and 1.0 (0.0–3.0), respectively.

### 2.3. Increased Numbers of Ly6G^+^ and Ly6C^+^ Cells Are Observed in the Ipsilateral Joints of SFN-Treated Mice

**Figure 3:**
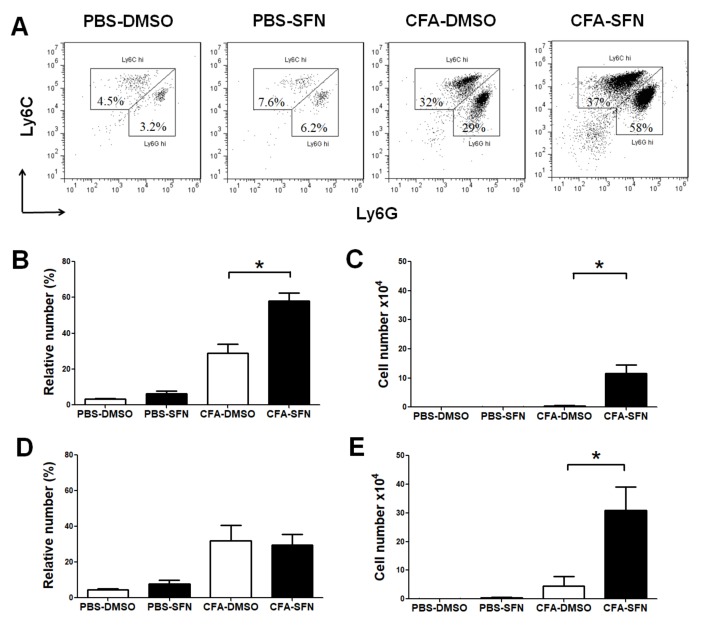
Phenotypic characterization of infiltrating cells in the knee joint of mice with mono-arthritis. Representative two-colour dot-plots for (**A**) Ly6G^+^ (neutrophils) and Ly6C^+^ (monocytes/macrophages) cells; relative (**B**) and absolute (**C**) numbers of Ly6G^+^ cells; and relative (**D**) and absolute (**E**) numbers of Ly6C^+^ cells in synovial fluid samples obtained from ipsilateral (CFA-injected) and contralateral (PBS-injected) joints of vehicle- (open bars) and SFN- (closed bars) treated mice. Data are mean ± SEM. Experiments were performed in four different occasions (*n* = 14 animals per group). * *p* < 0.05.

Treatment with SFN promoted the influx of neutrophils (Ly6G^+^) and macrophages (Ly6C^+^) to the ipsilateral (CFA-injected) but not contralateral (PBS-injected) knee joints (Figure 3A). Both the frequency (2-fold increase; Figure 3B) and numbers of Ly6G^+^ (33-fold increase; Figure 3C) cells were raised in the ipsilateral joints of SFN-treated in comparison with vehicle-treated mice. On the other hand, although SFN did not alter the frequency of Ly6C^+^ cells (Figure 3D) in the ipsilateral joint, this compound induced an increase in total number of infiltrating Ly6C^+^ cells (6.8-fold increase in comparison with vehicle group; Figure 3E).

### 2.4. SFN Decreases the Expression of CD11b and CD62L on Synovial Fluid Ly6G^+^ cells

**Figure 4:**
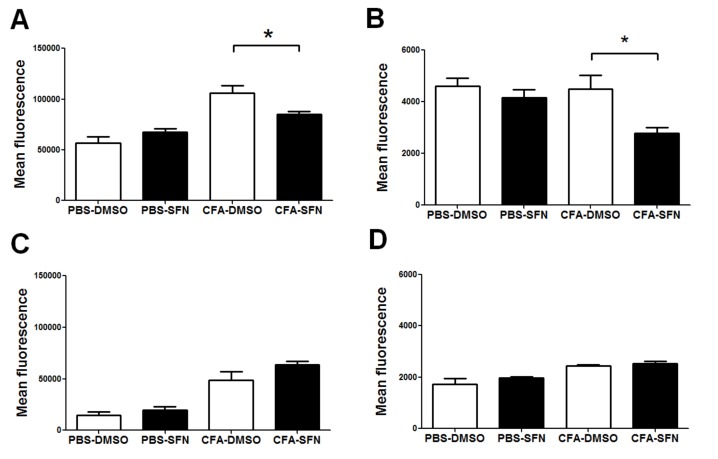
Expression of CD11b and CD62L on synovial fluid monocytes/macrophages and neutrophils. Expression of CD11b (**A**) and CD62L (**B**) on synovial fluid Ly6G^+^ cells. Expression of CD11b (**C**) and CD62L (**D**) on synovial fluid Ly6C^+^ cells. Samples were obtained from ipsilateral (CFA-injected) and contralateral (PBS-injected) joints of vehicle- (open bars) and SFN- (closed bars) treated mice. Data are mean ± SEM. Experiments were performed in four different occasions (*n* = 14 animals per group). * *p* < 0.05.

The administration of SFN down-regulated the expression of both CD11b (27%, Figure 4A) and CD62L (42%, Figure 4B) on synovial fluid Ly6G^+^ leukocytes in comparison with those of vehicle controls. However, SFN did not affect the expression of either CD11b or CD62L on monocytes/macrophages (Ly6C^+^) (Figure 4C,D, respectively).

### 2.5. SFN Increases the Levels of IL-6 in Plasma and Synovial Fluid Samples

**Figure 5:**
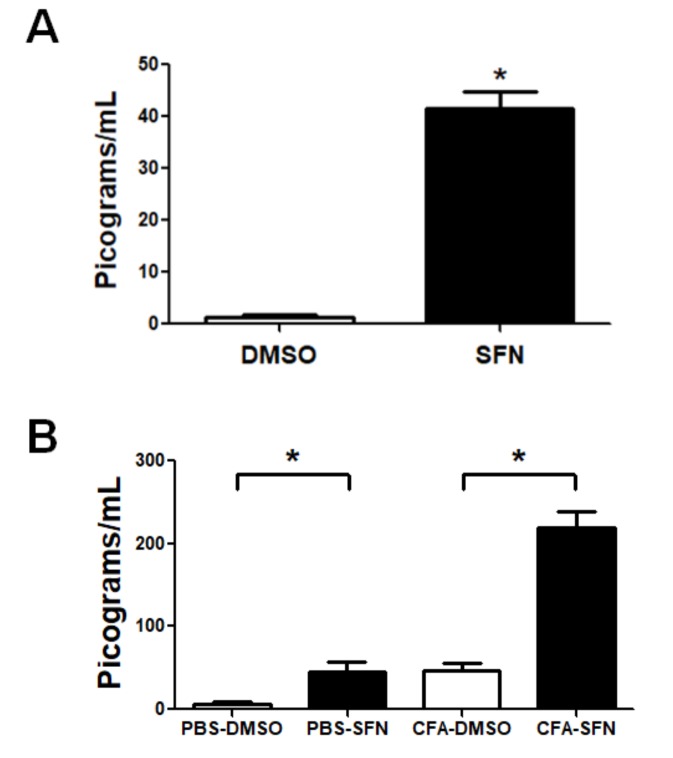
Circulating and synovial fluid levels of IL-6 in mice with mono-arthritis. Plasma (**A**) and synovial fluid (**B**) concentrations of IL-6 in samples obtained from vehicle- (open bars) and SFN- (closed bars) treated mice. Data are mean ± SEM. Experiments were performed in four different occasions (*n* = 14 animals per group). * *p* < 0.05.

Despite the seven cytokines tested, only IL-6 was detected. The plasma levels of IL-6 were significantly raised in animals treated with SFN in comparison with those that received vehicle (31.5-fold, Figure 5A). 

The same animals exhibited higher levels of this cytokine in synovial fluid samples obtained from their ipsilateral joints (CFA-injected; 4.5-fold increase). Interestingly, the levels of IL-6 in contralateral knees joints (injected with PBS) of animals treated with SFN were significantly higher (7-fold increase compared to vehicle-treated), at levels similar to those found in ipsilateral joints (CFA-injected) of vehicle-treated mice (Figure 5B).

### 2.6. TrxR Activity is Raised in Plasma and Synovial Fluid Samples of Mice with Joint Inflammation

**Figure 6:**
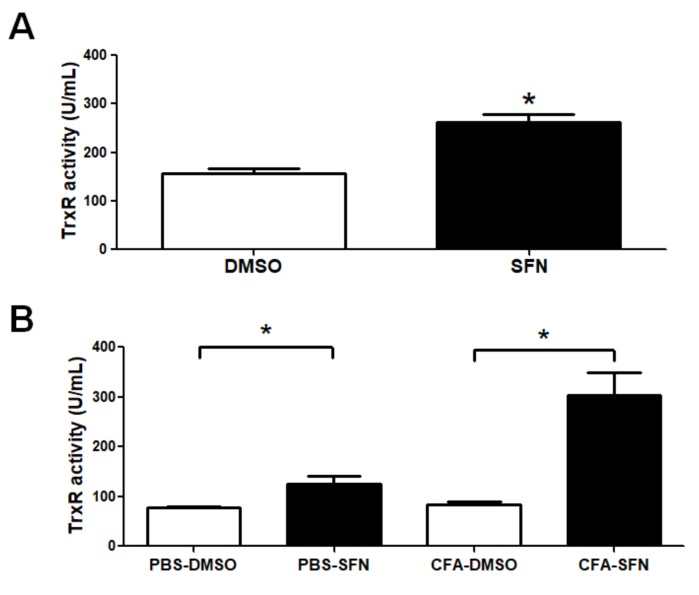
TrxR activity in plasma and synovial fluid samples of mice with mono-arthritis. Plasma (**A**) and synovial fluid (**B**) TrxR activity levels in samples obtained from vehicle- (open bars) and SFN- (closed bars) treated mice. Data are mean ± SEM. Experiments were performed in four different occasions (*n* = 14 animals per group). * *p* < 0.05.

TrxR activity was found to be increased in plasma samples obtained from mice treated with SFN (1.7-fold increase) in comparison with those treated with vehicle (Figure 6A). Likewise, SFN markedly augmented the activity of TrxR in the synovial fluid samples obtained from the ipsilateral (3.6-fold increase) and contralateral (1.6-fold increase) joints in comparison with those of vehicle-treated mice (Figure 6B).

## 3. Discussion

Sulforaphane (1-isothiocyanato-4-(methylsulfinyl) butane, SFN) is one the most extensively studied isothiocyanates, and is found in cruciferous vegetables such as broccoli and brussel sprouts; it has been reported to regulate chronic diseases, nephropathy, cancer and to reduce angiogenesis, oxidative stress and inflammation [18,19,20,21,22,23,31,32,33,34,35]. Here, to the best of our knowledge, we show for the first time that this compound increases neutrophil influx in to CFA-treated joints and down-regulates the expression of CD11b and CD62L on these cells. Furthermore, these effects are associated with increased production of IL-6 and enhanced activity of TrxR.

We found that the repeated treatment with SFN reduces knee joint inflammation/damage (denoted by data on knee joint thickness and histology) in animals with CFA-induced mono-arthritis, without affecting their nociceptive thresholds. On the other hand previous studies reported an anti-oedematogenic and anti-nociceptive effect of SFN [10,30,35], as demonstrated in a neuropathic pain model [36], where SFN was shown to reduce oedema, and mechanical allodynia and hyperalgesia. Differences between our data and the ones presented by these studies may be due to differences in the route of administration of the compound (intraperitoneal versus intrathecal), dose of the compound and/or length of treatment. These evidences suggest that SFN differently regulates peripheral and central events of joint inflammation.

The initial events of joint inflammation such as the associated with RA include an intense influx of inflammatory cells such as neutrophils and monocytes to the joints [5,6]. In this context, these cells have been linked to the aggravation of RA due to their ability to release cytotoxic molecules and ROS, causing exacerbation of inflammation [8]. We found that the repeated administration of SFN post-CFA injection increases the accumulation of Ly6C^+^ and Ly6G^+^ cells (indicative of neutrophils and mononuclear cells) in the synovial fluid [37]. Interestingly, Ly6G^+^ but not Ly6C^+^ cells exhibited less CD11b and CD62L on their surface. Whilst CD11b is a marker of leukocyte activation, loss of CD62L by shedding, down-regulates cell migration and activation during declining of inflammation [38]. It is is possible thus, that the inflammatory cell populations detected in the synovial fluid of the CFA-joints of SFN-treated animals are less activated than those of the vehicle group, positively impacting joint inflammation, by reducing knee joint thickness and intra-articular damage [39].

Oxidative stress normally occurs during inflammation and the thioredoxin system plays an important role in the control of excessive tissue injury. TrxR reduces oxidized thioredoxin (Trx), which in turn acts as an important removal tool of hydrogen peroxide and peroxynitrite, therefore protecting cells from damage and apoptosis [40,41]. Inhibition of TrxR activity results in accumulation of oxidized Trx triggering apoptosis [41,42]. Our data shows that SFN increases TrxR activity, suggesting a potential for this compound to attenuate cell damage in joint inflammation.

The same animals also presented with increased levels of IL-6 in their plasma and synovial fluid obtained from CFA joints. Released by both inflammatory and fibroblast-like synovial cells, IL-6 has a well-established role in the transition to chronicity and maintenance of joint inflammation, contributing to joint damage [43]. As mice treated with SFN exhibited higher levels of IL-6 but a smaller degree of joint inflammation, it is possible this cytokine was generated at a time point earlier than the one addressed herein and remained elevated, by mechanisms we could not yet determine. We suggest that the deleterious effects of the increased levels of IL-6 in SFN-treated mice may be overcomed by the increased activity of TrxR and attenuated activation of leukocytes in their CFA-joints. Of note, we were not able to detect other cytokines such as IL-2, IL-10, TNFα and IL-17 in plasma and synovial fluid samples in the time-point used herein. We cannot overrule possible effects of SFN in other time-points in which these cytokines are relevant for nociception and joint inflammation in the mono-arthritis induced by CFA.

## 4. Material and Methods

### 4.1. Animals

Non-fasted male C57BL/6 mice (6–8 weeks old) were used. Mice were obtained from the animal facility of the Uniceuma University (Sao Luis, Brazil) and were kept in a climatically controlled environment (room temperature of 22 ± 2 °C and humidity of around 60%) under a 12:12 h light-dark cycle (lights on at 7:00 a.m.). All procedures were approved by the Ethics Committee (protocol # 635072) of Uniceuma University and were carried out in accordance with the guidelines of the Society for Animal Welfare (SBCAL).

### 4.2. Complete Freund’s Adjuvant (CFA)-Induced Mono-Arthritis

For the induction of (CFA)-Induced Mono-Arthritis, the mouse knees were injected intra-articularly with 10 μg of CFA (10 μL in to the ipsilateral joint) and saline (0.9% sodium chloride, 10 μL in to the contralateral joint) [44]. Knee joint thickness and nociception were registered prior to and on the days 1, 3 and 7 post-CFA injection. Mechanical allodynia and hyperalgesia thresholds in the hind paws were determined using Von Frey filaments, as previously described [45,46]. Mice were placed in clear cages with a wire mesh floor and allowed to acclimatize for 30 min before the readings were taken. Measurements were obtained by repeated application (10 times on each paw) of 0.4 and 0.6 g force. The frequency of paw withdrawal was taken as indicative of allodynia and hyperalgesia, respectively. Results are expressed in percentage of paw withdrawal. Knee joint diameter was measured by calipers (Starrett, Sao Paulo, Brazil) and taken as an index of knee joint swelling. Results are expressed as the difference (Δ in mm) in knee joint thickness before and after the administration of CFA or vehicle.

### 4.3. Treatment with SFN

After induction of mono-arthritis by injection of CFA, six doses of SFN (10 mg/kg, Sigma Aldrich, Sao Paulo, Brazil, 10 mL/kg, *n* = 19) or vehicle (3% DMSO in saline, 10 mL/kg, *n* = 19) were administered intraperitoneally in each animal, one every 12 h, starting at 7:00 p.m. on the day 4 post-CFA injection. The last injection occurred at 7:00 a.m. on the 7th day, one hour before the measurements (allodynia, hyperalgesia and knee joint thickness) were taken.

### 4.4. Collection of Samples

On day 7 post-CFA induced mono-arthritis, animals received an overdose of a mixture of ketamine (75 mg/kg) and xylazine (1 mg/kg). Blood samples were then collected in heparinized tubes by cardiac puncture. Plasma was then separated by centrifugation and used for further analysis. Synovial fluid samples (*n* = 14/group) were collected with 0.5 mL micro-thin insulin syringes (BD, Sao Paulo, Brazil), by washing the intra-articular space 4 times with 25 µL of ice-cold PBS, resulting in 0.1 mL of sample. In a separate series of experiment, the knee joint samples (*n* = 5/group) were collected for histology on the day 7 post-CFA, just after the recording of nociceptive thresholds and knee joint thickness.

### 4.5. Cell Phenotype Characterization by Flow Cytometry

After lysis of red blood cells, total synovial fluid leukocytes were counted in a Neubauer chamber under microscopy (×10 objective). Single-cell suspensions were prepared, washed and resuspended in flow cytometry buffer ((2% foetal calf serum (Invitrogen, Sao Paulo, Brazil) in PBS (Sigma-Aldrich, Sao Paulo, Brazil), and stained with different monoclonal antibodies conjugated to fluorochromes, for 30 min: Ly6C and CD19 (FITC); Ly6G and CD3 (PE); CD11b and CD4 (PerCP); CD62L, CD14 and CD3 (APC), all from eBiosciences (Sao Paulo, Brazil). Data was collected in a BD Accuri C6 Flow Cytometer (BD Biosciences-Immunocytometry Systems, San Jose, CA, USA). The events were analysed with FlowJo 7.6.1 software (TreeStar-Ashland, OR, USA). Results are expressed as absolute cell counts or relative numbers; excepting those of CD62L and CD11b which were expressed as mean fluorescence.

### 4.6. Measurement of Cytokines

The levels of different cytokines (IL-2, IL-4, IL-6, IL-10, TNF-alpha, IFN-gamma and IL-17) were assessed in plasma and synovial fluid samples by cytometric bead array (CBA) with a Th1/Th2/Th17 kit (BD Biosciences, Sao Paulo, Brazil); according with the manufacturer’s instructions. Data collection and analysis were performed in a BD Accuri C6 flow cytometer. Results were calculated in CBA FCAP Array software (BD Biosciences, Sao Paulo, Brazil) and are expressed as picograms of cytokine per millilitre (pg/mL).

### 4.7. Thioredoxin Reductase (TrxR) Activity Levels

The activity levels of TrxR were evaluated in plasma and synovial fluid samples, by the reduction of 5′5-ditiobis 2-nitrobenzoic acid (DTNB). Briefly, 10 µL of each sample were incubated with 900 µL of a solution containing 0.24 mM NADPH, 10 mM EDTA, 100 mM potassium phosphate in ultrapure water; in the presence and absence of a specific trioredoxin reductase inhibitor, according with the manufacturer’s instructions (Sigma-Aldrich, Sao Paulo, Brazil). Thirty µL of DTNB (39.6 mg/mL) were added to each sample. TrxR (10 µg inTris-HCl 50 mM, pH 7.4, EDTA 1 mM) was used as control. Samples were incubated for 40 min and the reaction was then read at 405 nm. Results are expressed in units per millilitre (U/mL) as the difference in absorbance in the presence and absence of TrxR inhibitor.

### 4.8. Knee Joint Histology

The knee joint samples were collected, washed in PBS, infused with 10% formalin in PBS and then, processed for histology. Samples were decalcified by immersion in EDTA solution (4 °C for 14 days) and then were embedded in paraffin for cutting. Tissue samples were deparaffinized in xylene followed by dehydration in a graded series of ethanol/water. Serial 10 µm sagittal sections of the entire joint were cut on a microtome. Samples were stained with hematoxylin and eosin for observation of tissue morphology. A blinded observer (J.C.d.S.) then scored all sections for synovial hypertrophy, cell infiltration, cartilage destruction and bone erosion as previously described [44]. A 0–3-point scale was used for each parameter: synovial hypertrophy, cellular infiltration, cartilage destruction, bone erosion (0-normal, 1-mild, 2-moderate, 3-severe). The summation of the scores of each joint from each animal was used as index of joint inflammation/damage. Results are expressed as the median (minimum/maximum) of each group.

### 4.9. Statistical Analysis

The results are presented as the mean ± SEM. The percentages of inhibition are reported as mean ± SEM relative to the control samples. Samples were assessed for normal distribution by the Shapiro-Wilki’s test. Statistical comparisons of the data were performed by two-way analysis of variance (ANOVA) followed by Bonferroni’s test and unpaired *t* test when appropriate; except for histology. The results of the histologic evaluation are expressed as the median (minimum–maximum) in box plot graphs and were analyzed using Kruskal-Wallis followed by the Dunns test. *p* values < 0.05 were considered significant.

## 5. Conclusions

Overall, our data shows that the repeated administration of SFN protects against joint damage and inflammation caused by CFA, by increasing TrxR activity and regulating leukocyte activation and migration to the inflamed joint.

## References

[1] Schneider M., Kruger K. (2013). Rheumatoid arthritis—Early diagnosis and disease management. Dtsch. Arztebl. Int..

[2] Rantapaa-Dahlqvist S., de Jong B.A., Berglin E., Hallmans G., Wadell G., Stenlund H., Sundin U., van Venrooij W.J. (2003). Antibodies against cyclic citrullinated peptide and IgA rheumatoid factor predict the development of rheumatoid arthritis. Arthritis Rheumatol..

[3] Manzo A., Bombardieri M., Humby F., Pitzalis C. (2010). Secondary and ectopic lymphoid tissue responses in rheumatoid arthritis: From inflammation to autoimmunity and tissue damage/remodeling. Immunol. Rev..

[4] Catrina A.I., Joshua V., Klareskog L., Malmstrom V. (2016). Mechanisms involved in triggering rheumatoid arthritis. Immunol. Rev..

[5] Soler Palacios B., Estrada-Capetillo L., Izquierdo E., Criado G., Nieto C., Municio C., González-Alvaro I., Sánchez-Mateos P., Pablos J.L., Corbí A.L. (2015). Macrophages from the synovium of active rheumatoid arthritis exhibit an activin A-dependent pro-inflammatory profile. J. Pathol..

[6] Wipke B.T., Allen P.M. (2001). Essential role of neutrophils in the initiation and progression of a murine model of rheumatoid arthritis. J. Immunol..

[7] Sharma J., Bhar S., Devi C.S. (2017). A review on interleukins: The key manipulators in rheumatoid arthritis. Mod. Rheumatol..

[8] Ostrakhovitch E.A., Afanas’ev I.B. (2001). Oxidative stress in rheumatoid arthritis leukocytes: Suppression by rutin and other antioxidants and chelators. Biochem. Pharmacol..

[9] Hayer S., Bauer G., Willburger M., Sinn K., Alasti F., Plasenzotti R., Shvets T., Niederreiter B., Aschauer C., Steiner G. (2016). Cartilage damage and bone erosion are more prominent determinants of functional impairment in longstanding experimental arthritis than synovial inflammation. Dis. Mod. Mech..

[10] Bas D.B., Su J., Wigerblad G., Svensson C.I. (2016). Pain in rheumatoid arthritis: Models and mechanisms. Pain Manag..

[11] Efthimiou P., Kukar M. (2010). Complementary and alternative medicine use in rheumatoid arthritis: Proposed mechanism of action and efficacy of commonly used modalities. Rheumatol. Int..

[12] Efthimiou P., Kukar M., Mackenzie C.R. (2010). Complementary and alternative medicine in rheumatoid arthritis: No longer the last resort!. HSS J..

[13] Zhao S., Otieno F., Akpan A., Moots R.J. (2017). Complementary and Alternative Medicine Use in Rheumatoid Arthritis: Considerations for the Pharmacological Management of Elderly Patients. Drugs Aging.

[14] Colmegna I., Ohata B.R., Menard H.A. (2012). Current understanding of rheumatoid arthritis therapy. Clin. Pharmacol. Ther..

[15] Bykerk V.P., Akhavan P., Hazlewood G.S., Schieir O., Dooley A., Haraoui B., Khraishi M., LeClercq S.A., Légaré J., Mosher D.P. (2012). Canadian Rheumatology Association recommendations for pharmacological management of rheumatoid arthritis with traditional and biologic disease-modifying antirheumatic drugs. J. Rheumatol..

[16] Bornstein C., Craig M., Tin D. (2014). Practice guidelines for pharmacists: The pharmacological management of rheumatoid arthritis with traditional and biologic disease-modifying antirheumatic drugs. Can. Pharm. J..

[17] Dinkova-Kostova A.T., Talalay P. (2008). Direct and indirect antioxidant properties of inducers of cytoprotective proteins. Mol. Nutr. Food Res..

[18] Shin D.H., Park H.M., Jung K.A., Choi H.G., Kim J.A., Kim D.D., Kim S.G., Kang K.W., Ku S.K., Kensler T.W. (2010). The NRF2-heme oxygenase-1 system modulates cyclosporin A-induced epithelial-mesenchymal transition and renal fibrosis. Free Radic. Biol. Med..

[19] Srivastava R.K., Tang S.N., Zhu W., Meeker D., Shankar S. (2011). Sulforaphane synergizes with quercetin to inhibit self-renewal capacity of pancreatic cancer stem cells. Front. Biosci..

[20] Herman-Antosiewicz A., Johnson D.E., Singh S.V. (2006). Sulforaphane causes autophagy to inhibit release of cytochrome C and apoptosis in human prostate cancer cells. Cancer Res..

[21] Yoon H.Y., Kang N.I., Lee H.K., Jang K.Y., Park J.W., Park B.H. (2008). Sulforaphane protects kidneys against ischemia-reperfusion injury through induction of the Nrf2-dependent phase 2 enzyme. Biochem. Pharmacol..

[22] Zhao H.D., Zhang F., Shen G., Li Y.B., Li Y.H., Jing H.R., Ma L.F., Yao J.H., Tian X.F. (2010). Sulforaphane protects liver injury induced by intestinal ischemia reperfusion through Nrf2-ARE pathway. World J. Gastroenterol..

[23] Davis R., Singh K.P., Kurzrock R., Shankar S. (2009). Sulforaphane inhibits angiogenesis through activation of FOXO transcription factors. Oncol. Rep..

[24] Yu R., Lei W., Mandlekar S., Weber M.J., Der C.J., Wu J., Kong A.N. (1999). Role of a mitogen-activated protein kinase pathway in the induction of phase II detoxifying enzymes by chemicals. J. Biol. Chem..

[25] Guerrero-Beltran C.E., Calderon-Oliver M., Pedraza-Chaverri J., Chirino Y.I. (2012). Protective effect of sulforaphane against oxidative stress: Recent advances. Exp. Toxicol. Pathol..

[26] Hirota K., Nakamura H., Masutani H., Yodoi J. (2002). Thioredoxin superfamily and thioredoxin-inducing agents. Ann. N. Y. Acad. Sci..

[27] De Figueiredo S.M., Binda N.S., Nogueira-Machado J.A., Vieira-Filho S.A., Caligiorne R.B. (2015). The antioxidant properties of organosulfur compounds (sulforaphane). Recent Pat. Endocr. Metab. Immune Drug Discov..

[28] Petri S., Körner S., Kiaei M. (2012). Nrf2/ARE Signaling Pathway: Key Mediator in Oxidative Stress and Potential Therapeutic Target in ALS. Neurol. Res. Int..

[29] Prasad A.K., Mishra P.C. (2015). Mechanism of Action of Sulforaphane as a Superoxide Radical Anion and Hydrogen Peroxide Scavenger by Double Hydrogen Transfer: A Model for Iron Superoxide Dismutase. J. Phys. Chem. B.

[30] Kong J.S., Yoo S.A., Kim H.S., Kim H.A., Yea K., Ryu S.H., Chung Y.J., Cho C.S., Kim W.U. (2010). Inhibition of synovial hyperplasia, rheumatoid T. cell activation, and experimental arthritis in mice by sulforaphane, a naturally occurring isothiocyanate. Arthritis Rheum..

[31] Brandenburg L.O., Kipp M., Lucius R., Pufe T., Wruck C.J. (2010). Sulforaphane suppresses LPS-induced inflammation in primary rat microglia. Inflamm. Res..

[32] Zakkar M., Van der Heiden K., Luong le A., Chaudhury H., Cuhlmann S., Hamdulay S.S., Krams R., Edirisinghe I., Rahman I., Carlsen H. (2009). Activation of Nrf2 in endothelial cells protects arteries from exhibiting a proinflammatory state. Arterioscler. Thromb. Vasc. Biol..

[33] Megias J., Guillen M.I., Clerigues V., Rojo A.I., Cuadrado A., Castejon M.A., Gomar F., Alcaraz M.J. (2009). Heme oxygenase-1 induction modulates microsomal prostaglandin E synthase-1 expression and prostaglandin E(2) production in osteoarthritic chondrocytes. Biochem. Pharmacol..

[34] Kim J.K., Park S.U. (2016). Current potential health benefits of sulforaphane. EXCLI J..

[35] Wang C., Wang C. (2017). Anti-nociceptive and anti-inflammatory actions of sulforaphane in chronic constriction injury-induced neuropathic pain mice. Inflammopharmacology.

[36] Kim D., You B., Jo E.K., Han S.K., Simon M.I., Lee S.J. (2010). NADPH oxidase 2-derived reactive oxygen species in spinal cord microglia contribute to peripheral nerve injury-induced neuropathic pain. Proc. Natl. Acad. Sci. USA.

[37] Mayadas T.N., Cullere X. (2005). Neutrophil β2 integrins: Moderators of life or death decisions. Trends Immunol..

[38] Venturi G.M., Tu L., Kadono T., Khan A.I., Fujimoto Y., Oshel P., Bock C.B., Miller A.S., Albrecht R.M., Kubes P. (2003). Leukocyte migration is regulated by L-selectin endoproteolytic release. Immunity.

[39] Greco T., Shafer J., Fiskum G. (2011). Sulforaphane inhibits mitochondrial permeability transition and oxidative stress. Free Radic. Biol. Med..

[40] Holmgren A., Lu J. (2010). Thioredoxin and thioredoxin reductase: Current research with special reference to human disease. Biochem. Biophys. Res. Commun..

[41] Koháryová M., Kollárová M. (2015). Thioredoxin system—A novel therapeutic target. Gen. Physiol. Biophys..

[42] Tanito M., Masutani H., Kim Y.C., Nishikawa M., Ohira A., Yodoi J. (2005). Sulforaphane induces thioredoxin through the antioxidant-responsive element and attenuates retinal light damage in mice. Investig. Ophthalmol. Vis. Sci..

[43] McInnes I.B., Buckley C.D., Isaacs J.D. (2016). Cytokines in rheumatoid arthritis—Shaping the immunological landscape. Nat. Rev. Rheumatol..

[44] Fernandes E.S., Russell F.A., Spina D., McDougall J.J., Graepel R., Gentry C., Staniland A.A., Mountford D.M., Keeble J.E., Malcangio M. (2011). A distinct role for transient receptor potential ankyrin 1, in addition to transient receptor potential vanilloid 1, in tumor necrosis factor alpha-induced inflammatory hyperalgesia and Freund’s complete adjuvant-induced monarthritis. Arthritis Rheum..

[45] Quintao N.L., Medeiros R., Santos A.R., Campos M.M., Calixto J.B. (2005). The effects of diacerhein on mechanical allodynia in inflammatory and neuropathic models of nociception in mice. Anesth Analg..

[46] Bortalanza L.B., Ferreira J., Hess S.C., Delle Monache F., Yunes R.A., Calixto J.B. (2002). Anti-allodynic action of the tormentic acid, a triterpene isolated from plant, against neuropathic and inflammatory persistent pain in mice. Eur. J. Pharmacol..

